# Impact of the COVID-19 pandemic on pregnancy complications and conceptions resulting in births following spontaneous conception and in-vitro fertilization in British Columbia: A population-based study

**DOI:** 10.1371/journal.pone.0329683

**Published:** 2025-08-06

**Authors:** Rebecca Eckler, Bahi Fayek, Erica Won, Sabina Dobrer, Sarka Lisonkova, Paul J. Yong, KS Joseph, Mohamed A. Bedaiwy

**Affiliations:** 1 Department of Obstetrics and Gynecology, Faculty of Medicine, University of British Columbia, Vancouver, British Columbia, Canada; 2 Women’s Health Research Institute, Vancouver, British Columbia, Canada; 3 School of Population and Public Health, University of British Columbia, Vancouver, British Columbia, Canada; Kasr Alainy Medical School, Cairo University, EGYPT

## Abstract

**Objectives:**

To investigate the impact of the COVID-19 pandemic’s onset on clinical and demographic characteristics, pregnancy complications, and monthly conception rates resulting in births through spontaneous conceptions and in-vitro fertilization (IVF) in British Columbia (BC), Canada.

**Materials and methods:**

This retrospective population-based cohort study examined individuals who gave birth (both live births and stillbirths) in BC between June 1, 2010, and March 31, 2021, with estimated conception dates from January 1, 2010 to June 30, 2020. Data were obtained from the BC Perinatal Data Registry. Two groups were identified based on the estimated conception date: the pre-pandemic conception group (conception from January-1–2010 to February-29–2020) and the pandemic conception group (conception from March-1–2020 to June-30–2020). A time series forecasting method (ARIMA) was employed to observe trends in conception rates during the study period.

**Results:**

304,244 individuals with pregnancies resulting in live births or stillbirths during the study period were evaluated. A total of 429,843 such conceptions were included in the study: 417,753 (97.2%) occurring before the onset of the pandemic and 12,090 (2.8%) in the pandemic period. In June 2020, conceptions resulting in births decreased by 26.6% compared with June 2019. Trends in conceptions resulting in live births were similar, with a conception rate of 235.2 per 100,000 women of reproductive age in June 2020, compared with the expected rate of 305.0 per 100,000. In March 2020, 0.5% of conceptions that resulted in births ended in stillbirths, compared to 1.7% in March 2019. IVF conceptions ending in births declined during the pandemic, dropping to 2.5 and 2.8 per 100,000 in March and April 2020, respectively, compared with the expected 13.1 per 100,000. However, by June 2020, these rates began to recover toward the expected levels. Rates of gestational diabetes mellites (GDM), gestational hypertension (GHTN), and postpartum intensive care unit (ICU) admissions were similar before and during the COVID-19 pandemic.

**Conclusions:**

During the challenging period of the COVID-19 pandemic in BC, couples may have chosen to delay conception. Rates of conceptions resulting in stillbirths remained relatively unchanged. IVF conception rates were impacted by the suspension of elective procedures. Preterm birth rates slightly exceeded expected levels but remained within normal fluctuations, and small increases in GDM and GHTN prevalence were also observed.

## Introduction

Since the emergence of Severe Acute Respiratory Syndrome Coronavirus-2 (SARS-CoV-2) in late 2019, the virus and the pandemic response had devastating effects on health, societies, and economies around the world [1]. On March 11th, 2020, the World Health Organization declared the outbreak of SARS-CoV-2 as a global pandemic [2], and British Columbia (BC), Canada, recorded its first case of community transmission on March 5, 2020. Extensive research has explored the impact of the coronavirus disease-2019 (COVID-19) pandemic on pregnancy [3,4]. More investigations are required to comprehend its influence on conception and reproductive outcomes in pregnancies achieved through both spontaneous conception and assisted reproductive technologies (ART) such as in-vitro fertilization (IVF). COVID-19 pandemic-related restrictive measures included ART services. At the onset of the COVID-19 pandemic, reproductive medicine societies including American Society of Reproductive Medicine (ASRM), European Society of Human Reproduction and Embryology (ESHRE), International Federation for Fertility Societies (IFFS), and Canadian Fertility & Andrology Society (CFAS) recommended suspending non-urgent reproductive care, which resulted in a substantial reduction in the provision of infertility diagnoses, procedures, and treatments [5–7]. The COVID-19 pandemic has generated unique conditions that can impact conception rates. On one side, financial uncertainty related to reduced incomes and job losses, social crisis, and psychological impact of the COVID-19 pandemic have likely had effects on conception rates, particularly among infertile couples with reduced access to ART services [8]. On the other side, provincially mandated restrictive and self-quarantine measures at home might have led to a surge in conceptions and subsequent births, e.g., the “coronavirus baby boom”, similarly to the upticks in reproduction seen after wartime periods and blackouts [9,10]. In addition, during the 2020 lockdowns, limited access to family planning services and contraceptives due to manufacturing disruptions might have led to an increase in unplanned pregnancies and subsequent rise in birth rates [11]. As the COVID-19 pandemic situation improved, a careful, phased return to providing comprehensive reproductive services was approved [12]. Given all these factors, trends in conceptions after the pandemic onset that have led to births during the COVID-19 pandemic are not known. Thus, our study sought to 1) investigate shifts in clinical and demographic characteristics of women who conceived before and after the onset of the pandemic in BC, Canada; 2) evaluate the impact of the COVID-19 pandemic’s onset on pregnancy complications; and 3) explore changes in monthly conception rates leading to births through spontaneous conceptions and IVF conceptions across the onset of the COVID-19 pandemic.

## Materials and methods

### Study population

This retrospective population-based cohort study includes women aged 15–49 who gave birth (both live births and stillbirths) in BC between June 1, 2010, and March 31, 2021. The estimated conception dates for these births were between January 1, 2010, and June 30, 2020. We excluded conceptions resulting in pregnancy losses at < 20 weeks, as well as pregnancies with unknown birth dates, gestational ages at birth, or unspecified/unknown methods of conception (spontaneous conception versus IVF). Conceptions ending in births (for simplicity, further referred as conceptions) included in the study were categorized into two groups: pre-COVID-19 conceptions, which were achieved from January 1, 2010, to February 29, 2020, and COVID-19 conceptions, which conceived between March 1, 2020, and June 30, 2020. We examined maternal clinical and demographic characteristics by time of conception such as age, body mass index (BMI), parity, gravidity, self reported drug use during pregnancy, and self reported mental illness prior to or during pregnancy. Pregnancy complications including gestational diabetes mellites (GDM), gestational hypertension (GHTN), congenital fetal malformations (CFMFs), and postpartum intensive care unit (ICU) admissions were also investigated. We also conducted an analysis of conception rates resulting in live birth or stillbirth, comparing spontaneous versus IFV conceptions.

In addition, we analyzed clinical and sociodemographic characteristics, as well as pregnancy complications, for pre-COVID-19 conceptions during the pandemic compared to those of the total conceptions during the study period. This comparison aimed to determine whether this subset exhibited unique characteristics or experienced an increased frequency of pregnancy complications.

### Outcomes definitions

Gravidity was defined as the total number of pregnancies, including the current pregnancy, regardless of gestational age, type, timing, or method of termination/outcome [13]. Parity was defined as the number of previous pregnancies delivered at 20 or more completed weeks of gestation, or with a birth weight of 500 grams or more, regardless of the outcome [13]. Live births included all fetuses ≥20 weeks gestation showing signs of life at delivery, while stillbirths included all fetuses ≥20 weeks gestation who showed no signs of life at delivery [14]. Preterm birth was defined as the delivery of a live infant before completing 37 weeks of gestation [15]. GDM was diagnosed if the value of glucose challenge screening test is ≥ 11.1 mmol/L. Alternatively, the diagnosis could be made using a standardized 2-hour 75-g oral glucose tolerance test, if any one of the following values was met or exceeded: fasting plasma glucose of ≥5.1 mmol/L, 1-hour plasma glucose of ≥10.0 mmol/L, or 2-hour plasma glucose of ≥8.5 mmol/L [16]. GHTN was defined as systolic BP ≥ 140 mm Hg and/or diastolic BP ≥ 90 mm Hg, based on the average of at least 2 measurements that develop for the first time at ≥ 20 weeks of gestation, without evidence of preeclampsia [17]. Self-reported drug use during pregnancy included, but was not limited to, the use of heroin/opioids, cocaine, methadone, solvents, and marijuana at any time during the current pregnancy. Self-reported mental illness prior to or during pregnancy included, but was not limited to, depression, previous postpartum depression, anxiety, and bipolar disorder occurring prior to or during the current pregnancy. CFMFs encompassed malformations categorized in the ICD-10 codes (Q00-Q89), including those affecting the nervous system (Q00-Q07), eye, ear, face, and neck (Q10-Q18), circulatory system (Q20-Q28), respiratory system (Q30-Q34), cleft lip and cleft palate (Q35-Q37), digestive system (Q38-Q45), genital organs (Q50-Q56), urinary system (Q60-Q64), musculoskeletal system (Q65-Q79), and other congenital malformations (Q80-Q89) [18].

### Data source: BC Perinatal Data Registry (BCPDR)

De-identified data for this study were extracted, prepared, analysed, and accessed for research purposes over the period from 04-06-2022 to 01-03-2025 from the BC Perinatal Data Registry (BCPDR). BCPDR is a comprehensive provincial database that gathers data about obstetrical and neonatal outcomes. It includes more than 99% of births in BC, comprising deliveries in over 60 hospitals and at-home births overseen by registered midwives in the province. Since its inception in April 2000, this registry has records of more than 700,000 births [19]. The ethics approval for the study was obtained from the University of British Columbia Ethics Committee (H20-02316). Patient consent was waived for this study in accordance with the regulations of the UBC Research Ethics Board, as it involved a retrospective analysis of de-identified data.

### Determining conception rates

A birth-conception was defined as a conception that ultimately led to a recorded birth (whether live birth or stillbirth) in the BCPDR. The conception date was calculated using the newborn’s date of birth and the gestational age at delivery. The conception rate was calculated as the number of conceptions resulting in recorded births per 100,000 women of reproductive age (15–49 years old) in BC as of mid-June of each year.

### Statistical analysis

Summary statistics were utilized to describe clinical and demographic features characteristics, along with pregnancy complications. Categorical variables were summarized using the count and proportions of subjects in each category, while continuous variables were summarized using mean ± standard deviation or median and inter-quartile range.

We employed the Autoregressive Integrated Moving Average (ARIMA) model for time series forecasting [20,21] to assess temporal trends in clinical and demographic characteristics, pregnancy complications, and changes in the conception rates at the onset of the COVID-19 pandemic. The ARIMA model is characterized by its autoregressive, differencing, and moving average components. The Akaike Information Criterion and the Schwarz Bayesian Criterion were both used as model selection criteria (S1 Appendix). We utilized stratified ARIMA modeling to assess the impact of age and BMI on conception rates in the context of the COVID-19 pandemic.

### Additional analyses

We performed an age-period-cohort (APC) analysis, modified for gestational age. Since the information on the exact day of birth was unavailable, we randomly assigned a day within each birth month and estimated the day of conception by subtracting gestational age (in days). Conception cohorts were defined based on estimated day of conception, grouped into 14-days intervals to ensure an adequate number of births per cohort while maintaining relatively short intervals. Cumulative proportions of births in gestational age groups 30–31, 32–33, 34–35 weeks (preterm births) were plotted for each conception cohort, using the time of delivery as the x-axis. This approach allowed us to display conception cohorts that were conceived prior to the onset of the COVID-19 pandemic but delivered preterm during the pandemic, as well as those conceived at the onset of the pandemic and delivered in December 2021 and later. In addition, the proportion of births conceived via IVF was plotted for each conception cohort for gestational age intervals of 34–35, 36–37, 38–39, and 40–41 weeks, to ensure sufficient numbers within each gestational age group.

All statistical analyses were conducted using SAS Software version 9.4 (SAS Institute, Inc., Cary, NC, USA).

## Results

### Study population

**Fig 1:**
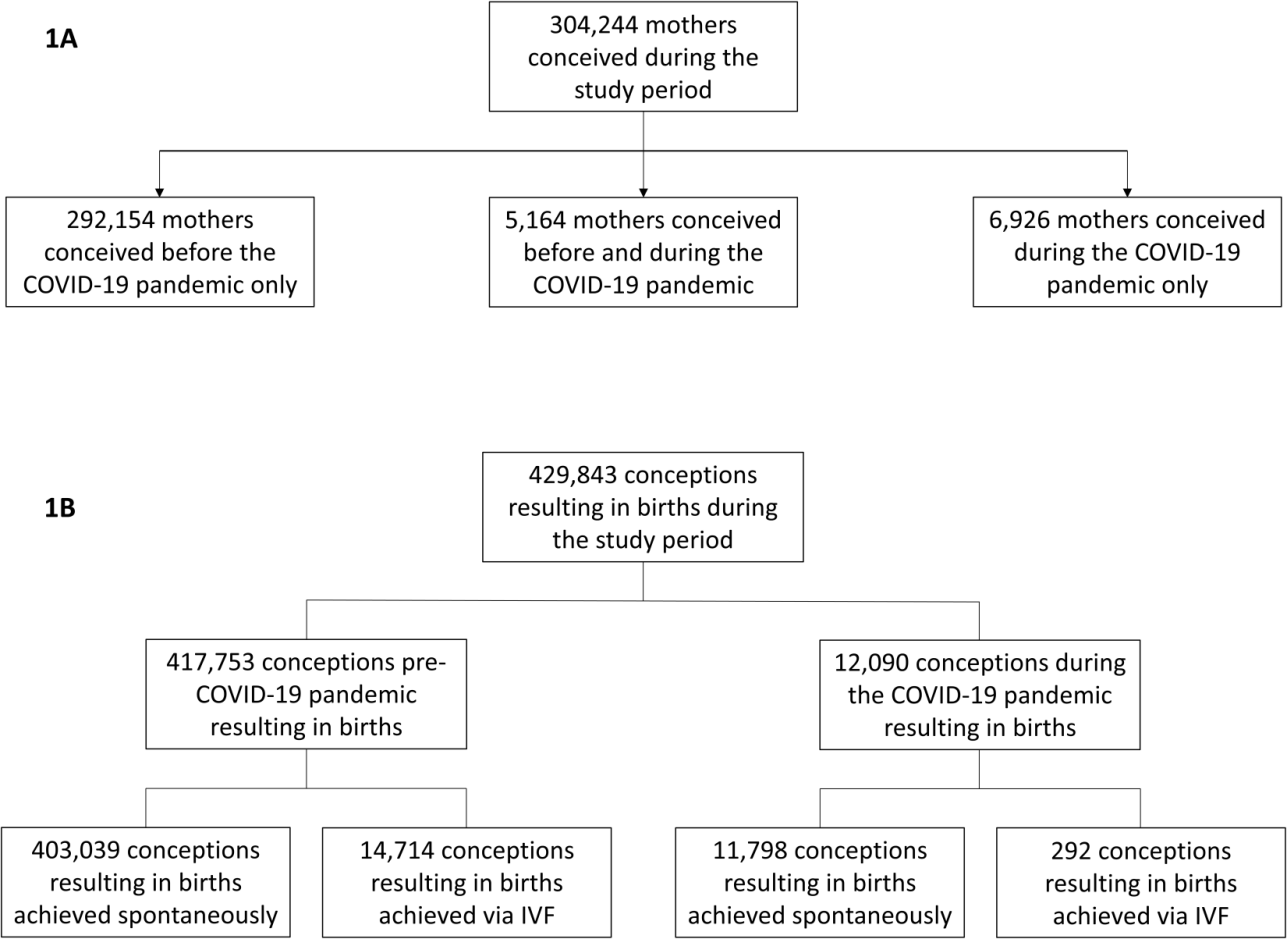
Flow chart of the studied population. (**A)** Flow chart of the included individuals; (**B)** Summary of conceptions resulting in birth during the study period (January 2010-June 2020). **Abbreviation:** IVF, In-vitro fertilization.

A total of 304,244 women had pregnancies resulting in live births or stillbirths during the study period: 292,154 (96.03%) conceived before the onset of the COVID-19 pandemic; 6,926 (2.28%) during the pandemic, and 5,164 (1.70%) conceiving during both time periods (women with subsequent pregnancies). These women achieved 429,843 conceptions during the study period: 417,753 (97.2%) occurring before the onset of the COVID-19 pandemic and 12,090 (2.8%) occurring during the pandemic. Among births conceived before the COVID-19 pandemic, 14,714 (3.5%) were achieved via IVF, whereas during the pandemic, only 292 conceptions (2.4%) were via IVF (Fig 1A and 1B). Out of the 417,753 conceptions occurring before the onset of the COVID-19 pandemic, 31,618 conceptions (7.5%) have resulted in birth during the COVID-19 pandemic.

### Clinical and demographic characteristics

The clinical and demographic characteristics by time of conception are presented in Table 1. There was a 1.9% increase in self-reported drug use during pregnancy in the COVID-19 conceptions (6.3%) compared with pre-pandemic conceptions (4.4%). During the COVID-19 pandemic, there was a 7.1% increase in self-reported mental illness prior to or during pregnancy compared with the pre-pandemic (28.2% versus 21.1%, respectively). Stratified ARIMA analysis showed that conception rates in women under 35 years were more impacted by the COVID-19 pandemic compared with those in women aged 35 year or older; as the difference between the observed and expected conceptions rates was 51.1 per 100,000 women of reproductive age among younger women, compared with 15 per 100,000 among older women by June 2020 (S1 Fig). Younger women may have chosen to delay conception due to greater reproductive timeline flexibility, whereas older women did not have the same opportunity. Conception rates in women with a normal BMI (18.5 to 24.9 kg/m^2^) were more impacted by the COVID-19 pandemic compared with those in women with underweight (BMI: < 18.5 kg/m^2^), overweight (BMI: 25–29.9 kg/m^2^), and obesity (BMI: ≥ 30 kg/m^2^). The differences between observed and expected conception rates were 10 times larger in normal BMI group compared with women with obesity (43.3 compared to 4 conceptions per 100,000 women of reproductive age by June 2020) (S2 Fig). The prevalence of self-reported mental illness prior to or during pregnancy showed minimal deviation from expected rates in the COVID-19 pandemic period (S3 Fig). Clinical and demographic characteristics of pre-COVID-19 conceptions resulting in births during the COVID-19 pandemic were not different from those of the total conceptions during the study period (S1 Table).

**Table 1:** Clinical and demographic characteristics and pregnancy complications of the studied population by time of conception.

Characteristics	Total 429,843 (100%)	Pre-COVID-19 conceptions 417,753 (97.2%)	COVID-19 conceptions 12,090 (2.8%)
**Age**			
Mean (SD)	31.51 (5.22)	31.49 (5.23)	32.22 (4.93)
**Pre-pregnancy BMI (Kg/m**^**2**^)			
Mean (SD)	24.62 (5.47)	24.59 (5.46)	25.27 (5.76)
Missing	93,582 (21.8%)	91,357 (21.9%)	2,225 (18.4%)
**Gravidity**			
Mean (SD)	2.37 (1.71)	2.37 (1.71)	2.35 (1.67)
Median (IQR)	2 (2)	2 (2)	2(2)
**Parity**			
Primipara	200,229 (46.6%)	194,494 (46.6%)	5,735 (47.4%)
Multipara	229,612 (53.4%)	223,257 (53.4%)	6,355 (52.6%)
**Gestational age at time of birth**			
Mean (SD)	38.46 (2.03)	38.47 (2.03)	38.12 (2.01)
**Self reported drug use during pregnancy**			
Yes	19,141 (4.5%)	18,378 (4.4%)	763 (6.3%)
No	410,702 (95.5%)	399,375 (95.6%)	11,327 (93.7%)
**Self reported mental illness prior or during pregnancy**			
Yes	91,736 (21.3.%)	88,324 (21.1%)	3,412 (28.2%)
No	338,107 (78.7%)	329,429 (78.9%)	8,678 (71.8%)
**Preterm birth**			
Preterm (<37 weeks)	41,253 (9.6%)	39,757 (9.5%)	1,496 (12.4%)
Full term (≥37 weeks)	388,586 (90.4%)	377,992 (90.5%)	10,594 (87.6%)
**Gestational hypertension**			
Yes	23,818 (5.5%)	22,924 (5.5%)	894 (7.4%)
No	406,025 (94.5%)	394,829 (94.5%)	11,196 (92.6%)
**Gestational diabetes mellites**			
Yes	51,973 (12.1%)	50,213 (12.0%)	1,760 (14.6%)
No	377,870 (87.9%)	367,540 (88.0%)	10,330 (85.4%)
Congenital fetal malformations*			
Yes	14,005 (3.3%)	13,493 (3.3%)	512 (4.3%)
No	415838 (96.7%)	404,260 (96.8%)	11,578 (95.8%)
**Postpartum ICU admissions**			
Yes	248 (0.1%)	242 (0.1%)	6 (0.1%)
No	429,595 (99.9%)	417,511 (99.9%)	12,084 (99.9%)

**Abbreviations:** BMI: Body mass index; ICU: Intensive care unit; SD: Standard deviation.

* Congenital fetal malformations included malformations categorized in the ICD-10 codes (Q00-Q89), including those affecting the nervous system (Q00-Q07), eye, ear, face, and neck (Q10-Q18), circulatory system (Q20-Q28), respiratory system (Q30-Q34), cleft lip and cleft palate (Q35-Q37), digestive system (Q38-Q45), genital organs (Q50-Q56), urinary system (Q60-Q64), musculoskeletal system (Q65-Q79), and other congenital malformations (Q80-Q89).

### Pregnancy complications

Pregnancy complications happening before and after the onset of the COVID-19 pandemic are presented in Table 1. There were no substantial differences in the proportions of GDM, GHTN, CFMFs, postpartum ICU admissions, and preterm births in conceptions before versus during the COVID-19 pandemic. These results have been confirmed by ARIMA modeling. The observed and expected prevalence of GDM in June 2020 were 16.5% versus 13.8%, respectively (S4 Fig). Likewise, the observed and expected prevalence of GHTN were 7.9% versus 6.5% in June 2020 (S5 Fig). There were no substantial differences in pregnancy complications between pre-COVID-19 conceptions resulting in births during the pandemic and those of the total conceptions during the study period (S1 Table).

### Conception rates

#### Conceptions resulting in births (live births and stillbirths).

**Fig 2:**
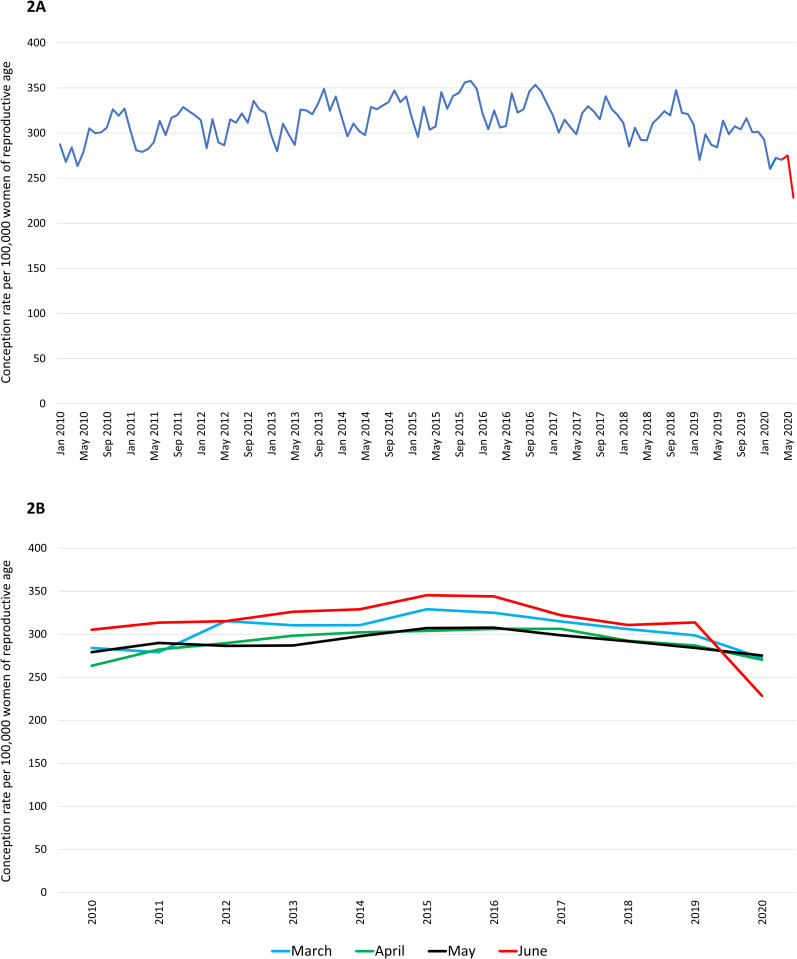
Conception rates resulted in births (live births and stillbirths) throughout the study period. (**A)**: Conception rates throughout the study period (January 2010-June 2020). (**B)**: Monthly conception rate trends from March to June across years (2010–2020).

**Fig 3:**
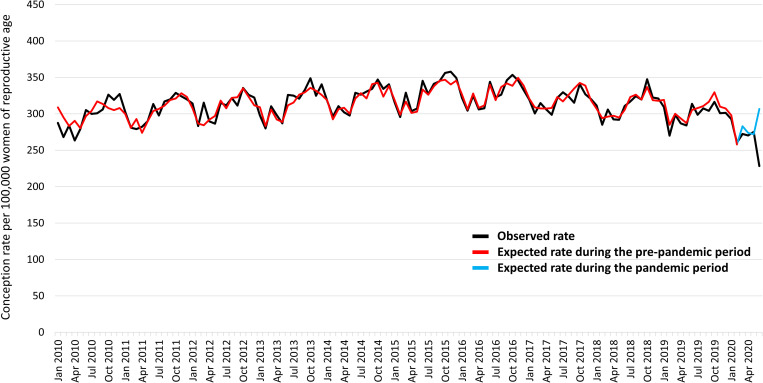
Observed and expected conceptions rates resulting in births (live births and stillbirths) during the study period.

Rates of conceptions resulting in births (live births or stillbirths) throughout the study period are presented in (Fig 2A and 2B). We observed seasonal variations in conceptions, characterised by lower rates in the months of February, higher rates in the months of October-December, and small peaks in the months of March and June of each year. Conception rates increased steadily from 2010 to 2015, then decreased gradually from 2015 to the beginning of 2020 to the levels comparable to 2010. ARIMA model-derived differences between observed and expected conception rates during the pre-pandemic period fluctuated between −27.1 and 31.2 conceptions per 100,000 women of reproductive age (Fig 3). Conception rates declined drastically in June 2020, far exceeding the seasonal patterns, with a 26.6% decrease compared with June 2019. The observed conception rate was 237.4 per 100,000 women of reproductive age compared with the expected conception rate of 306.7 (95% CI: 288.8–324.6) per 100,000 women of reproductive age in June 2020 (S2 Table).

#### Conceptions resulting in live births.

Similar trends were observed for conceptions resulting in live births, with an observed conception rate of 222.7 per 100,000 and expected conception rate of 299.3 per 100,000 women of reproductive age in June 2020 (95% CI: 281.6–317.0) (S2 Table).

#### Conceptions resulting in stillbirths.

To assess trends in conceptions resulting in stillbirths, we measured the proportion of conceptions resulting in stillbirths among all conceptions resulting in births rather than modelling stillbirths due to the inherently small numbers of stillbirths per month. The proportion of stillbirths among all births was very small during 2010−2020, fluctuating between 0.2% and 0.7%. During the COVID-19 pandemic, the proportion of conceptions resulting in stillbirths remained at similar levels, peaking at 0.5% in May 2020 (S3 Table).

#### Conceptions resulting in preterm births.

Observed preterm birth rates among live births exceeded the expected rates in pregnancies conceived in March, May, and June 2020, with differences in observed versus expected prevalence of 2.7, 1.8, and 5.1, respectively (S6 Fig).

#### IVF conceptions resulting in births.

**Fig 4:**
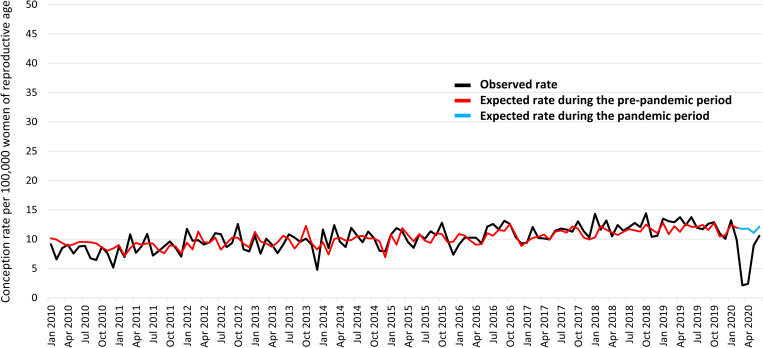
Observed and expected IVF conceptions rates resulting in births during the study period.

There was a decline in the observed IVF conception rates resulting in births with the onset of the COVID-19 pandemic (Fig 4). The observed rate was 9.9 per 100,000 women of reproductive age in February 2020, which then dropped to 2.1 per 100,000 in March 2020. However, it returned to normal expected rates by June 2020, reaching 10.6 per 100,000. Based on the ARIMA model, the expected IVF conception rate resulting in births was 11.7 per 100,000 in March 2020 and 12.1 per 100,000 in June 2020 (Fig 4).

### Additional analyses

APC plots showed that preterm birth at 32−33 and 33−34 weeks’ gestation was slightly elevated in March 2020 in cohorts conceived in the second half of April 2019 (S7 Fig). A similar increase was observed in cohorts conceived at the onset of the pandemic (March 2020), delivered in January 2021 (S7 Fig). However, these increases occurred within the range of temporal fluctuations in preterm birth rates at 32−33 and 34−35 weeks’ gestation. Additionally, the proportion of births conceived by IVF declined dramatically in cohorts conceived after the onset of the COVID-19 pandemic. This decline was observed at all gestational ages and was not accompanied by any apparent increase in preterm births (S8 Fig).

## Discussion

There are conflicting findings across various studies regarding the impact of the COVID-19 pandemic on conception rates leading to births and pregnancy complications. Several potential reasons can contribute to these discrepancies. First, differences in study populations and settings can significantly affect outcomes, as various regions experienced varying levels of pandemic-related disruption. Additionally, the timing of data collection plays a crucial role; the pandemic and pandemic-related restrictions had multiple phases with varying degrees of impact. In addition, studies employed various methodologies and used different data sources, including population data, hospital records, registries, and surveys, with different limitations and potential for a bias. Incomplete data collection, particularly in resource-limited settings or during peak pandemic periods, further complicates the validity and comparability of findings.

### Pregnancy complications

In our study, a slight increase in the observed rates of GDM was detected in June 2020 compared with expected rates. This slight increase may reflect the impact of pandemic lockdowns on lifestyle and stress. Changes in dietary habits, reduced physical activity due to the closure of recreational facilities, and increased stress from uncertainty, especially among pregnant individuals concerned about virus exposure and changes in healthcare access, may have led to excess weight gain and insulin resistance, increasing the risk of GDM [22]. Furthermore, during the COVID-19 pandemic, the Society of Obstetricians and Gynaecologists of Canada and Diabetes Canada recommended temporary alternative screening methods for GDM to reduce in-person clinical visits and minimize exposure risks [23]. These alternatives included using hemoglobin and/or random plasma glucose measurements instead of the standard oral glucose tolerance test [23]. This strategy aimed to identify individuals with significant hyperglycemia while minimizing exposure risks. Such changes in screening protocols may help explain the slight increase in GDM cases observed in June 2020. Several studies reported an increase in the prevalence or incidence of GDM during the COVID-19 pandemic period compared with pre-pandemic levels. A population-based study of 569,686 deliveries in Quebec, Canada, from 2014 to 2021, showed that during the first and second waves of the COVID-19 pandemic, GDM rates were higher compared with the pre-pandemic period. This increase was observed mainly among women considered at low risk of hyperglycemia who did not contract COVID-19 infections. The authors suggested that the sudden widespread changes in screening for GDM or lifestyle may have contributed to the rise in GDM rates [24]. A study in Northeast Italy compared GDM prevalence among women delivering during the COVID-19 pandemic (March 9th to May 18th, 2020) to those from 2019, finding a significant increase (9% versus 13.5%, p = 0.01). Experiencing lockdown during the first trimester was significantly associated with increased GDM incidence, resulting in a 34% monthly rise in diagnoses [25]. While GDM rates surged during the peak pandemic periods in some studies, a study observed a declining trend in GDM prevalence, diagnosed by oral glucose tolerance tests, from 2019 to 2021 following the initial pandemic impact (March 2020). Furthermore, pregnant women diagnosed with GDM during the most severe COVID-19 pandemic period did not face a higher risk of pregnancy complications compared with pre-pandemic times [26]. In our study, the observed prevalence of GHTN slightly exceeded the expected rates in May and June 2020, likely reflecting a combination of factors related to the COVID-19 pandemic. Similar to GDM, psychological stress is a known contributor to hypertension during pregnancy [27]. The lockdown period introduced substantial stressors, including increased anxiety about health, economic instability, and uncertainty surrounding the virus [28], may explain the increase in GHTN through the psychological toll of the COVID-19 pandemic. Additionally, the sedentary lifestyle associated with COVID-19 lockdown measures might have played a role in the slight increase in the observed rates of GHTN [29]. Moreover, disruptions to prenatal care during the early months of the COVID-19 pandemic, including reduced maternal healthcare visits and delayed prenatal screening [30], might have resulted in underreporting of GHTN cases initially, with a higher proportion subsequently identified in May and June 2020. In a retrospective analysis at four large hospitals comparing the COVID-19 pandemic period (April–July 2020) to the pre-COVID era (April–July 2019), involving 9,974 deliveries, a significant increase in HDP was observed during the pandemic (9.0% versus 6.9%; p < 0.01). This rise remained significant after adjusting for patient parity (odds ratio 1.41, 95% CI 1.20–1.66). However, no differences were noted in gestational age at delivery or the proportion of severe disease among women with HDP [31]. The observed upward trends in the prevalence of HDP during the COVID-19 pandemic may not necessarily denote a direct impact of the pandemic. Rather, they could signify a continuation of an existing trend established in previous years and persisting into the pandemic period. The global burden study, spanning from 1990 to 2019 (pre-pandemic), revealed a consistent rise in the incidence of HDP worldwide, with a total increase of 10.92% during this period [32].

### Conception rates

#### Conceptions resulting in live births.

The lower-than-expected conception rates resulting in live births observed in our study during the pandemic period could be attributed to concerns about the financial instability caused by the COVID-19 pandemic that might have influenced couples’ decisions regarding family planning [11]. The uncertainty about the long-term impacts of the pandemic might have also contributed to a decrease in birth conception rates [11]. Additionally, travel restrictions might have led to reductions in birth tourism (travel to Canada for birth) and prevented Canadian citizens living abroad who typically return to Canada for childbirth from doing so. Our findings are similar to previously conducted studies, indicating that the onset of the COVID-19 pandemic may have had an impact on the number of live births. McLaren et al found in a multicenter, retrospective study, a 19.8% decrease in live births reported at several hospitals in New York City and Long Island nine months after the pandemic-related lockdown measured in comparison with the previous year [33]. Similarly, Mendoza et al observed a notable reduction in live birth rates of 5.32% in Colombia in the first year of the pandemic compared with the mean national value for the past 6 years [34]. However, these studies did not account for temporal trends prior to the pandemic, which is a serious limitation.

#### Conceptions resulting in stillbirths.

Variations in stillbirth rates during the pandemic were evident across regions and populations, with reports of increased [35,36] and unchanged rates of stillbirth [37,38]. These differences may stem from variations in severity, pandemic-related restrictive measures, healthcare accessibility, differences in studied population, data sources, and socioeconomic factors. In our study, the proportion of conceptions resulting in stillbirths remained relatively unchanged during the onset of the COVID-19 pandemic. This stability in BC’s stillbirth rate can be attributed to the province’s swift public health response, which supported maternal-fetal health and enabled effective pregnancy management in the months following the onset of the COVID-19 pandemic. Our findings are in agreement with the second update of a living systematic review and meta-analysis, that showed no difference in the likelihood of stillbirth between the pandemic and pre-pandemic periods [37]. Similarly, Lisonkova et al found that the rates of stillbirth remained relatively stable in a conception cohort of non-IVF-conceived pregnancies conceived after the onset of the COVID-19 pandemic, while the stillbirth rate increased in those conceived by IVF [38]. There was no significant difference in stillbirth rates in Canada (excluding Québec) between March to August 2020 and the same period in 2019 [4]. Conversely, a population-based study of stillbirth rates in Canada and the United States of America (USA) from January 2015 to December 2020 revealed that in Canada, stillbirth rates were steadily rising before the pandemic, surged at its onset, and then sharply declined to levels lower than those observed pre-pandemic [39]. In contrast, the USA experienced a gradual decline in stillbirth rates before the pandemic followed by a sudden increase at the onset of the pandemic, then a sharp decline during the pandemic months, ultimately returning to pre-pandemic levels [39]. Nevertheless, all these studies examined rates of stillbirth during the first and subsequent months of the pandemic from pregnancies conceived prior to the pandemic. Our study provides a new perspective by examining the effects of the pandemic and pandemic-related measures as exposures during the time of conception, rather than at birth as in the majority of studies.

#### Conceptions resulting in preterm births.

We found that observed preterm birth rates among live births exceeded expected rates in conception occurring in March 2020 and continued to rise in those occurring in June 2020. However, these increases occurred within the range of temporal fluctuations in preterm birth rates at 32−33 and 34−35 weeks’ gestation. Molina et al observed that despite a 5.2% decline in total live births amid the COVID-19 pandemic, the prevalence of preterm births remained relatively unchanged at 10.7% throughout the study period, including both pre-pandemic and pandemic periods [40]. The preterm birth rates in Canada (excluding Québec) from March to August 2020 and in Ontario during the same period did not significantly differ from those in March to August 2019 [4]. In another large population study spanning Ontario’s first year of the COVID-19 pandemic (January-December 2020), the preterm birth rate (7.87%) was within the expected range based on nearly two decades of prior data (July 2002-December 2019) and did not show any unusual deviations that could be attributed to the pandemic itself [41]. However, a clear distinction should be drawn between the effects observed at the time of conception, as investigated in our study, and those at the time of birth, as documented in prior literature.

#### IVF conceptions resulting in births.

Our study reported declines in IVF rates at the onset of the pandemic, corresponding to the suspension of non-urgent reproductive services in March 2020. This was followed by a gradual return to pre-pandemic levels by June 2020, aligning with the easing of public health restrictions and resumption of fertility care. In an interrupted time series analysis of population-level data from USA comparing the expected and observed rates of ART-conceived live births and stillbirths among births conceived in March 2020 (delivering in November/December 2020) showed that in December 2020, there was a 57.0% decline in ART-conceived live births, while stillbirth rates increased among ART-conceived births during the same period [38]. In another IVF-clinic-based study conducted, 406 IVF cycles during the pandemic (COVID-19 group) were compared with 4197 IVF cycles before the pandemic (pre-COVID-19 group). The findings showed a significantly higher live birth rate in the COVID-19 group compared with the pre-COVID-19 group (51.4% versus 41.4%, P = 0.014) [42]. After 1:1 random matching, 403 cycles for each group were included in the study. The rates of fertilization, normal fertilization, and blastocyst formation were higher in the COVID-19 group than in the pre-COVID-19 group [42], however, this study did not account for pre-pandemic trends in these IVF-related outcomes. Additionally, in a single IVF center, comparisons between clinical pregnancy and live birth rates during the COVID-19 city lockdown period in 2020 (January 23 to February 23) and the same period in 2019 showed no significant differences for frozen embryo transfer. These findings suggest that COVID-19 did not have an adverse impact on IVF outcomes [43]. The contrasting trends observed between population-level data and data from IVF centers suggest that the decrease in IVF conception rates resulting in births at the population level may be attributed to restricted access to services rather than a direct effect of COVID-19.

### Strengths and limitations

Unlike institution-based studies prone to interpretation challenges from fluctuating outcome rates within healthcare facilities due to lockdown-triggered healthcare changes, the primary strength of this study lies in its population-based approach. The consistent data collection maintained by the BCPDR and a validation study showing high accuracy of pregnancy-related data are also strengths of this study [44]. The comprehensive inclusion of births across the province, including both hospital and home births, mitigates selection bias and increases generalizability. Additionally, the large population size, spanning an extensive timeframe, enhances our study’s statistical power. Another strength of this study is the use of birth cohort to evaluate pandemic influences confined to a specific period in calendar time by applying robust modeling for time series forecasting, known for its flexibility in handling a wide range of time series data. Our study has limitations. First, while our study provides valuable insights into the initial effects of the pandemic on conception trends in BC, it is essential to note that we did not capture the longer-term impacts as the available data only extends to pregnancies conceived prior to June 30, 2020. Second, our study examined conceptions resulting in births, which may not provide a comprehensive understanding of true conception rates during the study period, as it did not account for pregnancies that ended in pregnancy loss. Instead, we described the effects of pandemic-related exposures during the conception, conditional on the sustained pregnancy to 20 weeks’ gestation. Third, while our study highlighted disparities in IVF conceptions resulting in births before and after the onset of the COVID-19 pandemic, we lacked information on the use of other assisted reproductive techniques, such as controlled ovarian stimulation and intrauterine insemination. Fourth, changes in access to prenatal care during the early months of the pandemic may have impacted several conception cohorts, particularly those involving women who conceived before the pandemic but delivered during the pandemic. For instance, this could have influenced the detection and management of pregnancy complications such as GDM, which is typically diagnosed between 28 and 32 weeks of gestation. In addition, the use of information from large health databases means coding errors and omissions sometimes occur, and this may have increased during the first months of the COVID-19 pandemic. However, these errors would have likely resulted in non-differential misclassification affecting the descriptive characteristics of the cohorts, but not temporal trends. Finally, our study did not allow for an examination of how specific population subsets, such as those with differing socioeconomic status or residency (urban versus rural), were influenced by the pandemic in their decision to conceive and their access to assisted reproduction.

## Conclusions

The significant decrease in conceptions resulting in births in June 2020 highlights the influence of the COVID-19 pandemic, potentially reflecting couples’ decisions to postpone pregnancy during this challenging period. The proportion of conceptions resulting in stillbirths was unchanged during the onset of the COVID-19 pandemic reflecting an effective management of pregnancies to prevent stillbirth in later months after the pandemic onset. The suspension of diagnostic and elective procedures at the onset of the COVID-19 pandemic had an impact on IVF conception rates resulting in births, followed by a subsequent recovery to pre-pandemic levels by June 2020. Observed preterm birth rates slightly exceeded expected rates during the early COVID-19 pandemic but remained within the range of normal temporal fluctuations. Slight increases in the prevalence of GDM and GHTN were also observed during the COVID-19 pandemic, possibly reflecting the impact of lockdowns on lifestyle and stress.

## Supporting information

S1 Appendix ARIMA model. (DOCX)

S1 Table Clinical and demographic characteristics and pregnancy complications of pre-COVID-19 conceptions resulting in births (live births and stillbirths) during the COVID-19 pandemic. **Abbreviations:** BMI: Body mass index; ICU: Intensive care unit; SD: Standard deviation. **Legend:** * Congenital fetal malformations included malformations categorized in the ICD-10 codes (Q00-Q89), including those affecting the nervous system (Q00-Q07), eye, ear, face, and neck (Q10-Q18), circulatory system (Q20-Q28), respiratory system (Q30-Q34), cleft lip and cleft palate (Q35-Q37), digestive system (Q38-Q45), genital organs (Q50-Q56), urinary system (Q60-Q64), musculoskeletal system (Q65-Q79), and other congenital malformations (Q80-Q89).
(DOCX)

S2 Table Rates of observed and expected conception resulting in births (live births and stillbirths) during the COVID-19 pandemic. (DOCX)

S3 Table Proportion of conceptions resulted in stillbirth out of all conceptions resulted in births (live births and stillbirths) during Months March-June during the study period. **Legend:** * Rates per 100,000 women of reproductive age.
(DOCX)

S1 Fig Observed and expected conceptions rates in different age groups in regard to the COVID-19 pandemic onset. (TIF)

S2 Fig Observed and expected conceptions rates in regard to BMI and the COVID-19 pandemic. (TIF)

S3 Fig Observed and expected prevalence of self-reported mental illness prior to or during pregnancy during the study period. (TIF)

S4 Fig Observed and expected prevalence of gestational diabetes mellites during the study period. (TIF)

S5 Fig Observed and expected prevalence of gestational hypertension during the study period. (TIF)

S6 Fig Observed and expected conceptions rates resulting in preterm births among live births. (TIF)

S7 Fig Cumulative proportions of births at gestational ages 20–35 weeks (in 2-weeks intervals) for conception cohorts of fetuses conceived at the end of March 2019 and later that were delivered in the second half of November 2019 and later. **Legend:** Cohorts: the date of conception includes following next 14 days; calendar time dates include following 14 dates; dots represent gestational age weeks 20–21, 22–23, 24–25, 26–27, 28–29, 30–31, 32–33, 34–35 weeks. Shaded area represents the first months of the pandemic, March and April 2020; dashed lines represent conception cohorts conceived after the onset of the pandemic.
(TIFF)

S8 Fig Proportions of births conceived by IVF at gestational ages 34–35, 36–37, 38–39, 40–41 weeks (in 2-weeks intervals) for conception cohorts of fetuses conceived after 21 March 2019 who were delivered after 14 November 2019. **Legend:** Cohorts: the date of conception includes following next 14 days; calendar time dates include following 14 dates; dots represent gestational age weeks 34–35, 36–37, 38–39, 40–41 weeks. Shaded area represents the first months of the pandemic, March and April 2020; dashed lines represent conception cohorts conceived after the onset of the pandemic.
(TIFF)
