# Supplementary material for: Impact of the COVID-19 pandemic on pregnancy complications and conceptions resulting in births following spontaneous conception and in-vitro fertilization in British Columbia: A population-based study
Source: PLoS One. 2025 Aug 6;20(8):e0329683. doi: 10.1371/journal.pone.0329683 (PMC12327596; doi:10.1371/journal.pone.0329683)
Supplement: S1 Appendix — (DOCX) [file pone.0329683.s001.docx]

**S1 Appendix: ARIMA model**

The decision to use the ARIMA model was based on the stationarity of the time series as assessed through visual inspection and statistical tests, and differencing was unnecessary due to model stationarity. Stationarity was confirmed using Autocorrelation Check for White Noise with a p-value cut-off of 0.05. The model with the lowest Akaike Information Criterion and Schwarz Bayesian Criterion values was considered the best-fitting model as those values indicated a better trade-off between model fit and complexity. To assess potential autoregressive (AR) and moving average (MA) components, we utilized Autocorrelation Function and Partial Autocorrelation Function plots. Additionally, the Smallest CANonical correlation method was employed for the tentative identification of ARIMA process orders. We also applied the extended sample autocorrelation function method based on iterated least squares estimates of autoregressive parameters. Furthermore, we used the Minimum Information Criteria methods to determine the best combinations of AR and MA elements. To evaluate accuracy and performance of statistical models, residual rates calculated as the difference between observed and expected rates were assessed. The ARIMA model is praised for its simplicity, interpretability, and flexibility in handling various time series patterns, especially stationary data. It is well-supported by statistical theory and diagnostic tools, making it reliable for short to medium-term forecasting and widely trusted in various fields. Additionally, its integration with other methods and broad software availability enhances its practical utility for time series analysis. However, the ARIMA model assumes linearity and stationarity, which limits its effectiveness with non-linear and non-stationary time series. It requires a substantial amount of historical data for accurate parameter estimation and is sensitive to outliers and small sample sizes. Taking into consideration the population-level data in our study, large sample size, substantial historical data, short-term forecasting period, constant seasonality, and stationarity of the time series, together with rigorous procedures for choosing the AR and MA parameters and model validation techniques, played in favor of this specific modelling choice. Where we believed the ARIMA modelling performance was not the best, we used additional exploratory techniques to summarize the trends. One of the major limitations of ARIMA modelling is the inability to incorporate external variables, potentially limiting forecasting accuracy in complex datasets. However, stratification of the ARIMA modelling by various groups available in our study help us to identify specific trends for specific sub-populations.
