# Supplementary material for: Impact of the COVID-19 pandemic on pregnancy complications and conceptions resulting in births following spontaneous conception and in-vitro fertilization in British Columbia: A population-based study
Source: PLoS One. 2025 Aug 6;20(8):e0329683. doi: 10.1371/journal.pone.0329683 (PMC12327596; doi:10.1371/journal.pone.0329683)
Supplement: S1 Table — Abbreviations: BMI: Body mass index; ICU: Intensive care unit; SD: Standard deviation. Legend: * Congenital fetal malformations included malformations categorized in the ICD-10 codes (Q00-Q89), including those affecting the nervous system (Q00-Q07), eye, ear, face, and neck (Q10-Q18), circulatory system (Q20-Q28), respiratory system (Q30-Q34), cleft lip and cleft palate (Q35-Q37), digestive system (Q38-Q45), genital organs (Q50-Q56), urinary system (Q60-Q64), musculoskeletal system (Q65-Q79), and other congenital malformations (Q80-Q89). (DOCX) [file pone.0329683.s002.docx]

**S1 Table:** Clinical and demographic characteristics and pregnancy complications of pre-COVID-19 conceptions resulting in births during the COVID-19 pandemic

| **Characteristics** | **Total conceptions during the study period** **429,843** | **Pre-pandemic conceptions resulting in births during the COVID-19 pandemic (31,618)** |
| --- | --- | --- |
| **Age** | | |
| Mean (SD) | 31.51 (5.22) | 32.21 (4.99) |
| **Pre-pregnancy BMI (Kg/m^2^)** | | |
| Mean (SD) | 24.62 (5.47) | 25.05 (5.56) |
| Missing | 93,582 (21.8%) |  |
| **Gravidity** | | |
| Mean (SD) | 2.37 (1.71) | 2.34 (1.66) |
| Median (IQR) | 2 (2) | 2 (2) |
| **Parity** | | |
| Primipara | 200,229 (46.6%) | 15,112 (47.8%) |
| Multipara | 229,612 (53.4%) | 16,506 (52.20%) |
| **Gestational age at time of birth** | | |
| Mean (SD) | 38.46 (2.03) | 38.44 (1.86) |
| **Self reported drug use during pregnancy** | | |
| Yes | 19,141 (4.5%) | 1,772 (5.6%) |
| No | 410,702 (95.5%) | 29,846 (94.4%) |
| **Self reported mental illness prior or during pregnancy** | | |
| Yes | 91,736 (21.3.%) | 8,444 (26,71%) |
| No | 338,107 (78.7%) | 23,174 (73.29%) |
| **Preterm birth** | | |
| Preterm (<37 weeks) | 41,253 (9.6%) | 2,930 (9.33%) |
| Full term (≥37 weeks) | 388,586 (90.4%) | 28,688 (90.67%) |
| **Gestational hypertension** | | |
| Yes | 23,818 (5.5%) | 2,077 (6.57%) |
| No | 406,025 (94.5%) | 29,541 (93.43%) |
| **Gestational diabetes mellites** | | |
| Yes | 51,973 (12.1%) | 4,388 (13.88%) |
| No | 377,870 (87.9%) | 27,230 (86.12%) |
| **Congenital fetal malformations*** | | |
| Yes | 14,005 (3.3%) | 1,214 (3.84%) |
| No | 415838 (96.7%) | 30,404 (96.16%) |
| **Postpartum ICU admissions** | | |
| Yes | 248 (0.1%) | 19 (0,06%) |
| No | 429,595 (99.9%) | 31,599 (99.94%) |

**Abbreviations:** BMI: Body mass index; ICU: Intensive care unit; SD: Standard deviation

* Congenital fetal malformations included malformations categorized in the ICD-10 codes (Q00-Q89), including those affecting the nervous system (Q00-Q07), eye, ear, face, and neck (Q10-Q18), circulatory system (Q20-Q28), respiratory system (Q30-Q34), cleft lip and cleft palate (Q35-Q37), digestive system (Q38-Q45), genital organs (Q50-Q56), urinary system (Q60-Q64), musculoskeletal system (Q65-Q79), and other congenital malformations (Q80-Q89)
