# Supplementary material for: Impact of the COVID-19 pandemic on pregnancy complications and conceptions resulting in births following spontaneous conception and in-vitro fertilization in British Columbia: A population-based study
Source: PLoS One. 2025 Aug 6;20(8):e0329683. doi: 10.1371/journal.pone.0329683 (PMC12327596; doi:10.1371/journal.pone.0329683)
Supplement: S2 Table — (DOCX) [file pone.0329683.s003.docx]

|  | **COVID-19** **pandemic period** | **Observed conception rates** | **Expected**  **conception rates** | **Expected**  **95% CI** |
| --- | --- | --- | --- | --- |
| **Rates of conceptions resulting in births (live births and stillbirths)**  **per 100,000** **women of reproductive age** | Mar 2020 | 272.4 | 282.9 | 266.4-299.5 |
|  | Apr 2020 | 270.3 | 273.9 | 257.1-290.7 |
|  | May 2020 | 275.2 | 271.9 | 254.9-288.8 |
|  | Jun 2020 | 237.4 | 306.7 | 288.8-324.6 |
| **Rates of conceptions resulting in live births per 100,000 women of reproductive age** | Mar 2020 | 267.5 | 274.2 | 258.2-290.3 |
|  | Apr 2020 | 266.6 | 268.4 | 252.1-284.8 |
|  | May 2020 | 269.5 | 265.3 | 248.8-281.8 |
|  | Jun 2020 | 222.7 | 299.3 | 281.6-317.0 |
| **Rates of conceptions resulting in stillbirths per 100,000 women of reproductive age** | Mar 2020 | 0.51 | 1.14 | 0.37-1.91 |
|  | Apr 2020 | 1.19 | 1.19 | 0.42-1.96 |
|  | May 2020 | 1.28 | 1.28 | 0.49-20.7 |
|  | Jun 2020 | 0.77 | 1.29 | 0.49-2.08 |

**S2 Table:** Rates of observed and expected conception resulting in births (live births and stillbirths) during the COVID-19 pandemic:
