# Supplementary material for: Impact of the COVID-19 pandemic on pregnancy complications and conceptions resulting in births following spontaneous conception and in-vitro fertilization in British Columbia: A population-based study
Source: PLoS One. 2025 Aug 6;20(8):e0329683. doi: 10.1371/journal.pone.0329683 (PMC12327596; doi:10.1371/journal.pone.0329683)
Supplement: S3 Table — Legend: * Rates per 100,000 women of reproductive age. (DOCX) [file pone.0329683.s004.docx]

**S3 Table:** Proportion of conceptions resulted in stillbirth out of all conceptions resulted in births (live births and stillbirths) during Months March-June during the study period

|  | **Rates of conceptions resulted in births (live births and stillbirths)*** | **Rates of conceptions resulting in stillbirths*** | **Proportion of conceptions resulted in stillbirths out of all conceptions resulting in births (live births and stillbirths)** |  | **Rates of conceptions resulted in births (live births and stillbirths)*** | **Rates of conceptions resulted in stillbirths*** | **Proportion of conceptions resulted in stillbirths out of all conceptions resulted in births (live births and stillbirths)** |
| --- | --- | --- | --- | --- | --- | --- | --- |
| **March-2010** | 284.0 | 0.8 | 0.3 | **April-2010** | 263.4 | 1.3 | 0.5 |
| **March-2011** | 279.1 | 1.2 | 0.4 | **April-2011** | 282.2 | 1.6 | 0.6 |
| **March-2012** | 315.3 | 1.0 | 0.3 | **April-2012** | 289.6 | 1.0 | 0.3 |
| **March-2013** | 310.3 | 1.4 | 0.5 | **April-2013** | 298.2 | 1.4 | 0.5 |
| **March-2014** | 310.5 | 1.1 | 0.4 | **April-2014** | 302.1 | 1.5 | 0.5 |
| **March-2015** | 329.0 | 1.6 | 0.5 | **April-2015** | 303.8 | 1.8 | 0.6 |
| **March-2016** | 324.9 | 2.2 | 0.7 | **April-2016** | 306.1 | 1.5 | 0.5 |
| **March-2017** | 314.8 | 1.8 | 0.6 | **April-2017** | 306.2 | 1.2 | 0.4 |
| **March-2018** | 305.9 | 1.1 | 0.4 | **April-2018** | 292.4 | 1.2 | 0.4 |
| **March-2019** | 298.6 | 1.6 | 0.5 | **April-2019** | 286.9 | 0.9 | 0.3 |
| **March-2020** | 272.4 | 0.5 | 0.2 | **April-2020** | 270.3 | 1.2 | 0.4 |
| **May-2010** | 279.1 | 0.6 | 0.2 | **June-2010** | 305.1 | 1.3 | 0.4 |
| **May-2011** | 289.8 | 1.0 | 0.3 | **June-2011** | 313.4 | 1.6 | 0.5 |
| **May-2012** | 286.5 | 0.6 | 0.2 | **June-2012** | 315.1 | 0.7 | 0.2 |
| **May-2013** | 286.9 | 0.9 | 0.3 | **June-2013** | 326.0 | 1.3 | 0.4 |
| **May-2014** | 297.6 | 1.3 | 0.4 | **June-2014** | 328.9 | 1.0 | 0.3 |
| **May-2015** | 307.1 | 1.2 | 0.4 | **June-2015** | 345.3 | 1.3 | 0.4 |
| **May-2016** | 307.6 | 1.2 | 0.4 | **June-2016** | 343.9 | 0.9 | 0.3 |
| **May-2017** | 298.7 | 1.4 | 0.5 | **June-2017** | 322.1 | 0.7 | 0.2 |
| **May-2018** | 291.8 | 1.0 | 0.3 | **June-2018** | 310.7 | 0.9 | 0.3 |
| **May-2019** | 284.1 | 1.4 | 0.5 | **June-2019** | 313.7 | 1.2 | 0.4 |
| **May-2020** | 275.2 | 1.3 | 0.5 | **June-2020** | 228.3 | 0.8 | 0.4 |

* **Rates per 100,000 women of reproductive age**
